# Elektrotherapeutische Stellatumblockade bei einer Patientin mit komplexem regionalem Schmerzsyndrom der oberen Extremität

**DOI:** 10.1007/s00482-022-00682-6

**Published:** 2022-12-02

**Authors:** Alexander Ranker, Elke Behr-Eggers

**Affiliations:** 1 Klinik für Rehabilitationsmedizin, Medizinische Hochschule Hannover, Carl-Neuberg-Str. 1, 30625 Hannover, Deutschland; 2 Praxis für Allgemeinmedizin, Amelinghausen, Deutschland

**Keywords:** Physikalische Therapie, Hyperhidrose, Leitlinie, CRPS, TENS, Physical therapy, Hyperhidrosis, Guideline, CRPS, TENS

## Abstract

Berichtet wird von einer 51-jährigen Patientin mit komplexem regionalem Schmerzsyndrom („complex regional pain syndrome“ [CRPS]) der linken Hand nach Radiusdistorsion mit ossärer Fissur. Die antikonvulsive Therapie gestaltete sich bei bestehender Epilepsie mit bereits hoch dosierter dualer Therapie (Lamotrigin und Brivaracetam) schwierig. Bei bestehenden neuropathischen Schmerzen, ausgeprägter Allodynie und Hyperhidrose wurde eine repetitive transkutane monophasische Elektrotherapie über dem Ganglion stellatum angewandt. Eine Ganglionblockade konnte klinisch bei fehlendem Horner-Syndrom nicht bestätigt werden. Dennoch konnten neuropathischer Schmerz und Hyperhidrose positiv beeinflusst werden. Dieser Fallbericht fasst die verwendeten Elektrodenpositionen, Stromparameter, Fallstricke sowie Therapielimitationen zusammen und diskutiert diese mit der Literatur.

## Abstract (en)

The case of a 51-year-old patient with complex regional pain syndrome (CRPS) of the left hand after radius distortion is reported. Anticonvulsant therapy was difficult in this case due to persisting epilepsy with already dual therapy (lamotrigine and brivaracetam) at high dosage. With existing neuropathic pain, pronounced allodynia and hyperhidrosis, repetitive transcutaneous monophasic electrotherapy was applied above the stellate ganglion. A ganglion blockage could not be clinically confirmed in the absence of Horner syndrome, but neuropathic pain and hyperhidrosis could be positively influenced. This case report summarizes the electrode positions used, current parameters, pitfalls and therapy limitations and discusses them in relation to the literature.

## Einleitung

Die Blockade des Ganglion stellatum ist laut S1-Leitlinie „Diagnostik und Therapie komplexer regionaler Schmerzsyndrome“ [1] die Ultima Ratio bei der Therapie des komplexen regionalen Schmerzsyndroms (CRPS) der oberen Extremität mit schwacher Evidenz [2]. Dabei ist sowohl das Risikoprofil zu beachten als auch die Tatsache, dass zur Durchführung einer Stellatumblockade sowohl Intubationsbereitschaft als auch sterile Bedingungen vorliegen müssen. Das macht die Stellatumblockade zu einem invasiven Verfahren, welches nur großen Schmerzzentren vorbehalten ist.

Eine alternative Möglichkeit zur Reduktion der sympathischen ganglionären Aktivität ist die elektrische Nervenblockade. Diese wurde bereits von Jenkner Anfang der 80er-Jahre beschrieben [3, 4] und zeichnet sich durch ein sehr geringes Risikoprofil aus, was sie besonders für ambulante Schmerztherapien im niedergelassenen Sektor interessant macht.

**Figure 1:**
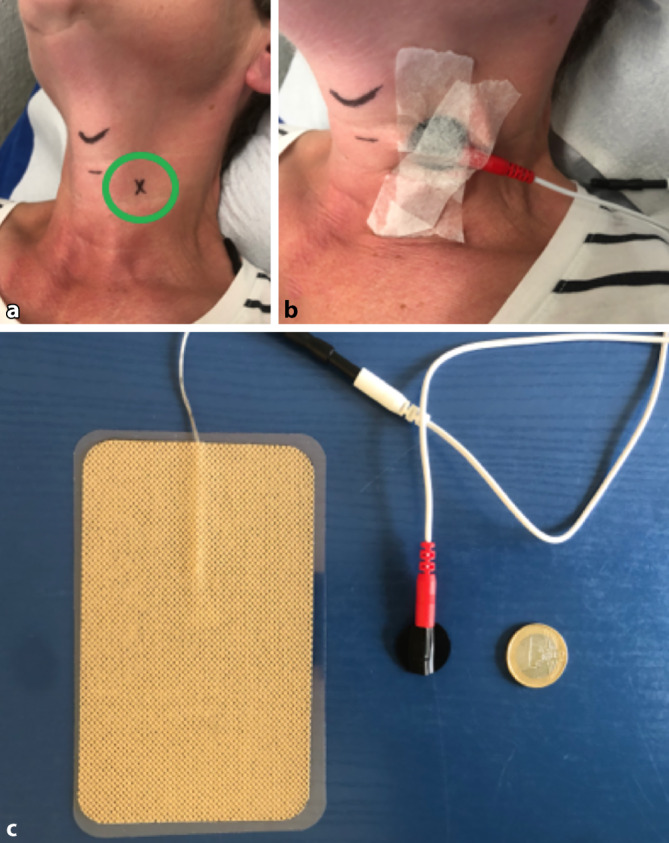

Es handelt sich dabei um monophasischen Strom (Gleichstrom), der im Sinne einer Galvanisation zu einer Hyperpolarisation der Nervenmembran führt [3–5]. Dieser Effekt wird durch Erhöhung der Felddichte noch verstärkt, indem man eine sehr kleine Elektrode als Anode (sog. Pierenblock) und eine große Elektrode als Kathode (siehe Abb. 1) verwendet [3, 5]. Eine Analgesie im Nervengebiet sowie eine Reduktion der sympathischen Aktivierung des Ganglions durch eine solche Therapie wurden postuliert [6].

Faktisch handelt es sich um eine transkutane elektrische Nervenstimulation (TENS). Es wird jedoch kein biphasischer Reizstrom wie bei der klassischen TENS verwendet und die empfohlene Frequenz ist mit 20–35 Hz tiefer [3, 4]. Das Frequenzspektrum der klassischen TENS liegt bei 50–150 Hz [7].

Trotz guter Ergebnisse von Jenkner selbst (*immer* ein Horner-Syndrom [3]) und einer bestehenden Empirie ist die Evidenzlage hinsichtlich einer echten Blockade schlecht und so zu werten, dass es nicht möglich oder zumindest unwahrscheinlich ist, mittels transkutaner Nervenstimulation eine echte Stellatumblockade auszulösen [8, 9].

Dennoch können Nervenfasern transkutan beeinflusst werden. Besonders bei repetitiver Anwendung wurden positive Ergebnisse gezeigt [10], auch im Hinblick auf die Reduktion von Hyperhidrose der Hände [11]. Zudem ist neben einer Hyperämisierung mit keiner weiteren Folge oder starken Nebenwirkungen zu rechnen. Allerdings ist zu beachten, dass im Gegensatz zur biphasischen Elektrotherapie (z. B. klassische TENS) Metallimplantate im Stromgebiet bei einer Galvanisation eine Kontraindikation darstellen [7].

## Anamnese und klinische Untersuchung

Frau M. ist eine 51-jährige Pflegefachkraft mit Zustand nach Sturz auf die rechte Hand. Eine unfallchirurgische Vorstellung resultierte in einer orthetischen Immobilisation des Handgelenks (Handgelenksorthese mit dorsopalmarer Fixierung in 0°) und Ibuprofen 600 mg 1‑1‑1 bei radiologisch gesicherter ossärer Fissur des Radius und Frakturausschluss. Acht Wochen nach Sturz erfolgte die ambulante schmerztherapeutische Erstvorstellung in unserer Praxis. Anamnestisch hätten nach 4‑wöchiger Ruhigstellung sukzessive Folgesymptome begonnen, mit Verdacht auf ein beginnendes CRPS. Die Patientin berichtete von anhaltendem Schmerz mit Veränderung der Schmerzqualität in Richtung neuropathischer Schmerzen, veränderter Hauttrophik mit Seitenasymmetrie des Hautkolorits, vermehrtem Haarwachstum auf dem Handrücken, starkem Schwitzen palmar sowie von zunehmenden motorischen Einschränkungen in Daumenflexion und Adduktion. Eine ausgeprägte Allodynie am Thenar, am gesamten Daumen und Zeigefinger sowie über dem distalen Radius wurde ebenso berichtet. Letzteres sei laut Frau M. am meisten einschränkend in der Partizipation am täglichen Leben. Zudem berichtete die Patientin von dumpfen Schmerzen in der Tiefe des Gelenks, die durch Temperaturwechsel und Aufregung gesteigert würden.

Die Schmerzintensität wurde in Ruhe mit 4/10 NRS („numeric rating scale“), min. 2/10 NRS und max. 8/10 NRS, beschrieben. Schmerzlindernd sei Ruhe. Schmerzverstärkend seien Belastung, psychische Anspannung sowie Temperaturwechsel (z. B. aus der Wohnung ins Freie gehen). Als Vorerkrankung ist neben arterieller Hypertonie, Hypothyreose (substituiert euthyreote Stoffwechsellage) und rezidivierenden depressiven Episoden (leichtgradig) besonders die Grand-mal-Epilepsie nennenswert. Diese sei durch eine duale antikonvulsive Therapie in hoher Dosis (Lamotrigin 400 mg/d und Brivaracetam 100 mg/d) gut eingestellt und die Patientin sei seit über einem Jahr anfallsfrei. Allerdings wurden kognitive Einschränkungen aufgrund der Medikation seitens der Patientin beschrieben, die sich in Konzentrationsstörungen und Vergesslichkeit äußern würden. Sozialanamnestisch lebe die Patientin mit ihrem Ehemann zu Hause, sei gelernte Pflegefachkraft, arbeite aber in Teilzeit als Aushilfskraft in einer Hausarztpraxis. Psychosoziale Belastungsfaktoren hinsichtlich Arbeitsbelastung sowie interfamiliärer Spannungen und monetärer Sorgen wurden beschrieben.

Die Diagnosestellung erfolgte nach Kriterien der International Association for the Study of Pain (IASP; [12]). Inspektorisch auffällig waren ein leichtes Ödem des Handgelenks sowie des Handrückens und eine im Seitenvergleich sichtbar hellere, teilweise marmorierte und livid erscheinende Haut. Die sensomotorische Untersuchung erfolgte nach Akklimatisierung der Patientin an das Raumklima mit standardisierter Untersuchung im Seitenvergleich. Die Hauttemperatur wurde mittels Infrarotthermometer an 4 Stellen gemessen (zwei Stellen dorsal, zwei Stellen palmar). Im Schnitt zeigte sich eine Seitendifferenz von −1,2 °C (die betroffene Seite imponierte kälter). Die Greiffunktion der rechten Hand war deutlich eingeschränkt (Pinzettengriff rechts insuffizient mit Fingerabstand Dig I–Dig II 2 cm; Faustschluss insuffizient mit einem Fingerkuppen-Hohlhand-Abstand von 2,5 cm). Die „range of motion“ im Handgelenk war mit Pronation/Supination 40°-0°-20° reduziert und endgradig schmerzhaft. Bei der Sensibilitätsüberprüfung fielen eine lokale Kälteallodynie sowie eine mechanische Allodynie palmar im Bereich des Daumengrundgelenks bis etwa 3 cm proximal des Radiokarpalgelenks palmarseitig auf. Die Messung des Handgelenkumfangs zeigte eine Umfangsvergrößerung rechts zu links von 2 cm. Gemäß Budapest-Kriterien wurde die Diagnose eines CRPS Typ I gestellt [12–14].

Als therapeutische Strategie wurde ein multimodales Konzept gewählt und die Patientin ausführlich beraten [13]. Es wurde Physiotherapie zur Kompensation pathologischer Bewegungsmuster verordnet. Dabei wurde explizit auf der Heilmittelverordnung vermerkt, auch im schmerzhaften, aber tolerierbaren Bereich zu üben (im Sinne einer „pain exposure physical therapy“). Zusätzlich erhielt die Patientin dreimal wöchentlich Ergotherapie (sensorisch-perzeptive Behandlung zur Desensibilisierung sowie Spiegeltherapie). Aufgrund des Ödems wurde eine Kortisonstoßtherapie (Prednisolon 80 mg, abdosiert innerhalb von zwei Wochen) initiiert. Bei der Wiedervorstellung nach zwei Wochen zeigten sich ein Rückgang des Ödems (Seitenunterschied nur noch < 1 cm im Handgelenkumfang) und eine geringfügig verbesserte Greiffunktion (Finger-Hohlhand-Abstand 1 cm, Pinzettengriff Dig I–II 0 cm, Dig I–Dig III 0,5 cm). Der tiefe Dauerschmerz hätte sich etwas reduziert.

Weiterhin vorherrschend wären anamnestisch allerdings die allodynischen Beschwerden sowie die palmare Hyperhidrose. Eine antikonvulsive Therapie mit Gabapentin wurde in einschleichender Dosis versucht, musste jedoch schon bei 600 mg/d (300 mg 1‑0-1) aufgrund nicht tolerierbarer kognitiver Einbußen mit Konzentrationsschwierigkeiten, Müdigkeit und subjektiv empfundener mentaler Abwesenheit abgebrochen werden. Nach interdisziplinärer Rücksprache mit der behandelnden Neurologin war eine Reduktion der bestehenden Antiepileptika nicht empfehlenswert. Im Konsens mit der Patientin wurden alternative physikalische Therapiemöglichkeiten zur Symptomlinderung der Allodynie und Hyperhidrose evaluiert.

Hydroelektrische Bäder (Zweizellenbad), die als Heilmittel verordnet werden können und ebenso ein Gleichstromverfahren sind [7], wurden schmerzbedingt nicht toleriert. Daraufhin wurde dann eine probatorische elektrische monophasische Stimulation über dem Ganglion stellatum durchgeführt. Im Gegensatz zu den bestehenden Studien [3, 4, 8] wurde hier jedoch die therapeutische Idee verfolgt, täglich repetitive elektrotherapeutische Behandlungen durchzuführen. Es wurde angenommen, dass dadurch summativ schmerzreduzierende und sympathikotonussenkende Effekte erzielt werden könnten. Zudem könnten die Selbstverantwortung und Compliance der Patientin gestärkt werden.

## Methoden

Verwendet wurde ein handelsübliches TENS-Gerät (TENStem eco basic, Fa Schwa-medico, Ehringshausen, Deutschland) mit einem monophasischen Programm (Galvanisation, Programm 16). Das Aufsuchen des Ganglion stellatum wurde sowohl ultraschallgestützt durchgeführt als auch nach den klassischen Landmarken (ca. 3 cm lateral und 3 cm kranial der Fossa jugularis im tastbaren Sulcus zwischen Trachea und M. sternocleidomastoideus in Höhe des Ringknorpels [C6], Abb. 1). Über dem Ganglion wurde mittels Ultraschallgel die kleine Anode (Pierenblock, Durchmesser 20 mm, Abb. 1) sowie dorsal über Halswirbelkörper 6 (HWK 6) bis Brustwirbelkörper 3 (BWK 3) die große Kathode (130 mm × 80 mm) aufgeklebt, um sicherzugehen, dass sich das Ganglion stellatum im Stromflussgebiet befindet. Anschließend erfolgte die erste Therapie unter ärztlicher Aufsicht und Monitoring (EKG, Pulsoxymeter, Blutdruck). Es wurde ein monophasischer Rechteckimpuls mit einer Frequenz von 35 Hertz (Hz) verwendet. Dieser wurde in 1 Milliampere(mA)-Schritten erhöht. Ab einer Stromintensität von 8 mA konnte eine tonische Kontraktion des M. omohyoideus sowie von Teilen des Platysmas beobachtet werden, die als sehr unangenehm beschrieben wurde. Daher wurde auf 7 mA reduziert. Auch minimale Positionsänderungen des Pierenblocks konnten eine tonische Kontraktion nicht verhindern. Somit waren 7 mA als Therapieintensität definiert.

Der Anlagepunkt des Pierenblocks wurde mit einem wasserfesten Stift auf der Haut aufgezeichnet. Die Patientin wurde aufgefordert, die TENS mit dieser Einstellung täglich zweimal im häuslichen Setting anzulegen (20 min je Therapiesitzung). Die Elektrotherapie wurde additiv zu der bereits beschriebenen physikalischen Therapie durchgeführt.

## Ergebnisse

Bereits bei der ersten Anwendung zeigte sich eine milde Hyperämisierung um den Pierenblock. Frau M. berichtete von einem Ziehen im gesamten Arm. Ein Horner-Syndrom blieb aus. Die Schmerzintensität des tiefen Schmerzes im Bereich des Radiokarpalgelenks hätte sich nach 20 min Therapie merklich reduziert (initial 6/10 NRS auf 3/10 NRS). Eine Überprüfung der Allodynie mittels Kältereiz und taktiler Reizung zeigte keinen merklichen Unterschied zur Ausgangslage.

Die Reevaluation erfolgte im wöchentlichen Abstand. Frau M. berichtete kontinuierlich von reduzierter Allodynie und weniger tiefen, dumpfen Schmerzen. Die Schmerzen würden unmittelbar bei der Therapie weniger werden und der Effekt würde etwa zwei Stunden anhalten. Die 7 mA konnten jedoch nie erhöht werden. Offenbar zeigte sich kein Gewöhnungseffekt.

Acht Wochen nach primärer Anwendung der Elektrotherapie zeigte sich eine deutliche Reduktion der Allodynie in der klinischen Untersuchung. Frau M. tolerierte nun auch Wasserbäder gut, sodass additiv Kohlensäurebäder rezeptiert wurden [15].

Eine radiologische Kontrolle zeigte eine beginnende Demineralisierung im Bereich des distalen Radius. Eine Bisphosphonattherapie wurde jedoch seitens der Patientin abgelehnt. Die Physiotherapie wurde intensiviert. Die Hyperhidrose war im Alltag nicht mehr spürbar, dennoch gab es selten Momente von plötzlicher starker Schweißsekretion palmar (z. B. ausgelöst durch starke Temperaturdifferenz beim Verlassen der Wohnung ins Freie).

## Diskussion

Die elektrische Stellatumblockade nach Jenkner konnte in mehreren „randomized controlled trials“ (RCT) nicht bestätigt werden [6, 7]. Larsen B. et al. führten bereits 1995 eine randomisierte, kontrollierte Studie an *n* = 50 Probanden durch, mit wahlweise monophasischem Strom nach den postulierten Parametern nach Jenkner oder gewöhnlicher biphasischer TENS mit einer Frequenz von 100 Hz [6]. Es zeigte sich kein signifikanter Unterschied zwischen den Gruppen, weder in der Hauttemperatur noch in der Schmerzintensität. Ebenso konnte bei keinem der Probanden ein Horner-Syndrom gezeigt werden. Bestätigt wurden diese Ergebnisse von Vacariu G. et al. [7]. An *n* = 12 gesunden Probanden wurde eine TENS im Gebiet um das Ganglion stellatum durchgeführt, wobei hier ein biphasischer Strom verwendet wurde. Es konnte kein signifikanter Unterschied in der Schmerzschwelle detektiert werden und kein Horner-Syndrom beobachtet werden [7].

Im vorliegenden Fall kam es ebenfalls zu keiner echten sympathischen Blockade des Ganglion stellatum, was durch ein Horner-Syndrom als Indikator bestätigt werden würde. Dennoch konnte eine Schmerzreduktion erzielt werden und subjektiv auch die Hyperhidrose deutlich reduziert werden.

Ob jedoch die vorliegende Schmerzreduktion durch die Elektrotherapie bedingt war oder auch ohne Elektrotherapie im Sinne einer zeitbezogenen Selbstlimitierung stattgefunden hätte, kann nicht beantwortet werden. Zusätzlich sollte beachtet werden, dass simultan weitere physikalische Therapien stattgefunden haben. Die Ergotherapie, im Sinne einer sensorisch-perzeptiven Behandlung zur Desensibilisierung und Spiegeltherapie, fand zwei- bis dreimal pro Woche statt. Zusätzlich erhielt die Patientin noch Physiotherapie (manuelle Therapie) mit der gleichen Frequenz. Medikamentös wurde mittels täglicher Etoricoxibeinnahme eine dauerhafte selektive Inhibition der Cyclooxygenase‑2 (COX2) durchgeführt, die ebenso auf die Verbesserung der Symptomatik Einfluss haben könnte. Zusammenfassend ist es eben die multimodale Therapie, die bei einem CRPS als zielführend gesehen werden kann und leitliniengemäß empfohlen werden sollte [1].

In diesem Zusammenhang ist zu betonen, dass das Risikoprofil einer transkutanen Elektrotherapie sehr günstig ist und sie somit ein hilfreicher additiver Baustein in diesem multimodalen Therapiesetting sein kann. In einem übersichtlichen editorialen Review zu elektrotherapeutischen Stellatumblockaden schlussfolgert Ammer K., dass eine schmerzreduzierende Wirkung von Elektrotherapie unbestritten sei [6]. Dies kann durch diesen Fall bestätigt werden.

Zudem kann dadurch die Patientencompliance gefördert werden und die Eigeninitiative und Selbstverantwortung geschärft werden. Die aktive Rolle und Selbstverantwortung des Patienten ist für die Akzeptanz des Schmersyndroms wichtig. Das Bewusstwerden der Selbstwirksamkeit durch eine konkrete Aufgabe, wie beispielsweise die Selbstanlage des TENS-Geräts, kann dazu beitragen, dass der Patient sich selbst als aktiven Teil der Schmerztherapie wahrnimmt.

Im vorliegenden Fall kann durchweg positiv von der Verwendung der transkutanen Elektrotherapie um das Stellatumgebiet gesprochen werden, wobei keinerlei allgemeingültige Therapieempfehlungen daraus gezogen werden können.

Diskrepanz herrscht bei der zu verwendenden Intensität. In der Literatur wird eine Stromstärke bis 35 mA empfohlen [5, 11], wobei im vorliegenden Fall bereits ab 8 mA die ventrale Halsmuskulatur tonisch reagierte. Die Patientin hat einen sehr schlanken Hals mit wenig Unterhautfettgewebe, was diesen Unterschied eventuell erklären könnte (Abb. 1), allerdings wären dann trotzdem deutlich geringere Stromstärken appliziert worden als laut Literatur üblich [5, 11].

## Fazit für die Praxis

Dieser Fall zeigt, dass in der Therapie des CRPS eine häusliche, tägliche therapeutische Nervenbeeinflussung mittels monophasischer TENS als additive physikalische Therapie sinnvoll sein kann. Schmerzen können beeinflusst werden, Eigenverantwortung und Selbstmanagement können gestärkt werden. Dabei ist das Risikoprofil der Therapie äußerst gering und sie kann in einem ambulanten Setting (z. B. Praxis) erprobt werden.
